# Beyond ER Stress: The Pleiotropic Roles of XBP1 in Development and Regeneration

**DOI:** 10.3390/biomedicines13112663

**Published:** 2025-10-30

**Authors:** Delan Huang, Fan Gu, Jingzhi Ma, Zhi Chen

**Affiliations:** 1 Department of Stomatology, Tongji Hospital, Tongji Medical College, Huazhong University of Science and Technology, Wuhan 430030, China; huangdl@tjh.tjmu.edu.cn; 2 State Key Laboratory of Oral & Maxillofacial Reconstruction and Regeneration, Key Laboratory of Oral Biomedicine Ministry of Education, Hubei Key Laboratory of Stomatology, School & Hospital of Stomatology, Wuhan University, Wuhan 430079, China; gufan1994@whu.edu.cn; 3 Department of Cariology and Endodontics I, Hospital of Stomatology, Wuhan University, Wuhan 430079, China

**Keywords:** XBP1, development, tissue homeostasis, ER stress, regeneration

## Abstract

This review synthesizes current knowledge on the roles of X-box binding protein 1 (XBP1) in development and regenerative medicine. XBP1 is defined as a key transcription factor that regulates biological processes from embryogenesis to adult tissue homeostasis via both endoplasmic reticulum(ER) stress-dependent and independent mechanisms. Evidence for its regulatory role in cell fate determination and tissue maintenance across multiple systems is presented. The therapeutic potential of targeting XBP1 is explored, particularly for the regeneration of skeletal muscle, skin, and bone. Critical future research priorities are outlined, such as deciphering the precise functions of the Inositol requiring enzyme 1 (IRE1α)/XBP1 signaling axis and evaluating the long-term safety of its modulation. XBP1 is thus confirmed as a prime target for advancing developmental biology and pioneering new regenerative therapies.

## 1. Introduction

The precise regulation of developmental processes and the extraordinary capacity for tissue regeneration represent fundamental biological phenomena with far-reaching implications for both basic science and clinical medicine [1,2]. These processes are governed by intricate molecular networks and regulatory mechanisms that coordinate cell fate determination, tissue homeostasis, and regenerative potential [3,4]. At the core of these networks, individual transcription factors exhibit context-dependent regulatory versatility—they can bind to multiple genomic loci, while the cis-regulatory function of enhancers requires combinatorial binding of diverse transcription factors [5]. This cooperative binding paradigm enables genes to receive sophisticated spatiotemporal-specific regulation. Among the key transcription factors, X-box binding protein 1 (XBP1) has emerged as a critical focus of recent research due to its multifaceted roles in development and regeneration [6,7].

**Figure 1:**
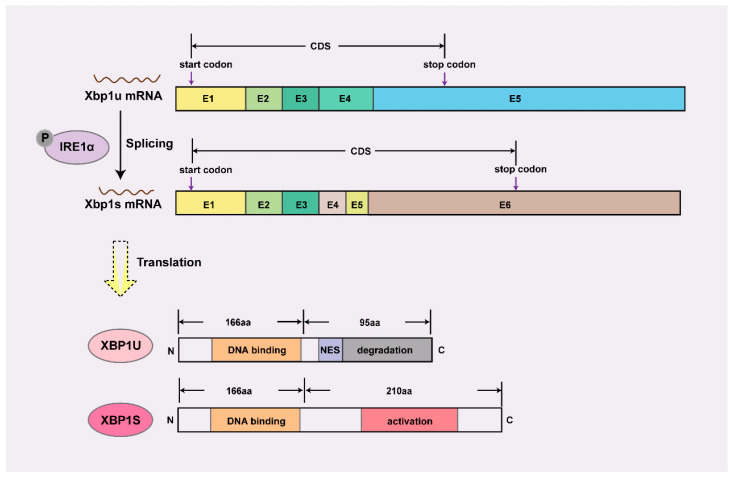
Schematic diagram of mRNA and protein structure of *Xbp1*. XBP1U retains a DNA-binding domain but is sequestered in the cytoplasm by its C-terminal nuclear export signal (NES) and targeted for proteasomal degradation via a degradation domain, rendering it transcriptionally inactive. In contrast, XBP1S acquires a C-terminal transcriptional activation domain through alternative splicing, enabling nuclear localization and target gene regulation, thus serving as the dominant functional isoform. E, exon; CDS, coding sequence; P, phosphorylation; N, amino terminus; C, carboxy terminus.

XBP1 is a highly conserved mammalian transcription factor belonging to the basic leucine zipper (bZIP) family. As a nodal regulator, XBP1 operates at multiple hierarchical levels—from orchestrating early lineage commitment through direct transcriptional control [6], to maintaining proteostasis *via* its canonical unfolded protein response (UPR) function during rapid tissue expansion [8,9]. The *Xbp1* gene undergoes unique post-transcriptional splicing that generates two functionally distinct isoforms (Figure 1), illustrating how alternative splicing converts the latent XBP1U isoform into the potent transcriptional activator XBP1S [10].

This review synthesizes current knowledge on XBP1’s pleiotropic functions in developmental biology, with particular emphasis on its molecular mechanisms of action; tissue-specific regulatory networks; and translational potential in regenerative medicine. Through critical analysis of existing literature, we aim to provide a conceptual framework for understanding XBP1’s integrative roles in development and its emerging therapeutic applications.

## 2. The Regulatory Role of XBP1 in Organogenesis and Tissue Homeostasis

**Figure 2:**
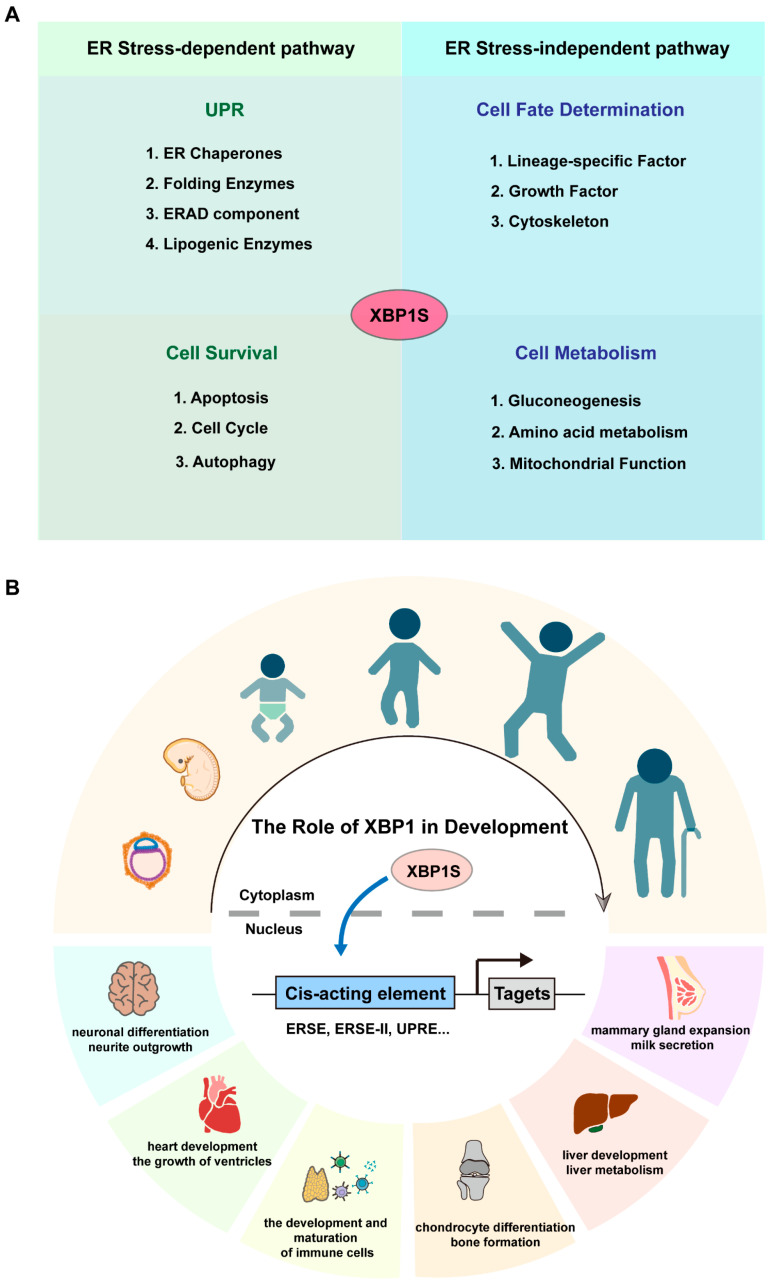
The role of XBP1 in the development. (**A**) Schematic representation of XBP1’s regulatory mechanisms. ER, endoplasmic reticulum; ERAD, ER-associated degradation. (**B**) Schematic representation of XBP1’s developmental roles, showcasing its impact on processes such as neural differentiation, heart ventricle growth, immune cell development, chondrocyte differentiation, mammary gland function, and liver metabolism. ERSE, Endoplasmic Reticulum Stress Response Element; ERSE-II, Endoplasmic Reticulum Stress Response Element-II; UPRE, Unfolded Protein Response Element.

While early investigations primarily focused on XBP1’s canonical role in UPR-mediated stress responses, emerging evidence suggests more diverse functions in development and tissue maintenance that extend beyond its classical ER stress-related activities (Figure 2A) [8,11,12]. As illustrated in Figure 2B, XBP1 orchestrates key developmental processes including neural differentiation, heart ventricle growth, immune cell development, chondrocyte differentiation, mammary gland function, and liver metabolism. Mechanistically, XBP1 operates through two parallel pathways: during organogenesis, it predominantly utilizes ER stress-independent mechanisms to direct lineage specification (e.g., neuronal commitment, osteoblast maturation), except in secretory tissues where ER biogenesis is obligatory (Table 1). Conversely, tissue homeostasis relies principally on its ER stress-dependent activity, particularly in combating proteotoxicity in neurodegeneration and maintaining metabolic balance (Table 2).

The selection between ER stress-dependent and independent activation of XBP1 is governed by a spectrum of contextual cellular cues. Key among these is the nature and intensity of extrinsic stimuli—for instance, varying degrees of viral infection or metabolic stress can preferentially engage one pathway over the other. Furthermore, cell-type-specific signaling environments significantly influence this choice; immune cells may utilize different regulatory networks compared to neurons, even under similar stimuli. Another crucial layer of regulation arises from crosstalk with other major signaling pathways, such as NF-κB, HIF-1α, and autophagy, which can directly or indirectly modulate XBP1 activity. Notably, certain plasma membrane receptor signaling pathways can activate UPR sensors, including IRE1α, independently of classical ER stress, thereby engaging XBP1 in a non-canonical manner [36]. Additionally, post-translational modifications of XBP1 and its interactors (e.g., phosphorylation, ubiquitination) and epigenetic landscapes that determine target gene accessibility provide further fine-tuning, collectively ensuring a precise and context-appropriate cellular response.

### 2.1. Nervous System

**Figure 3:**
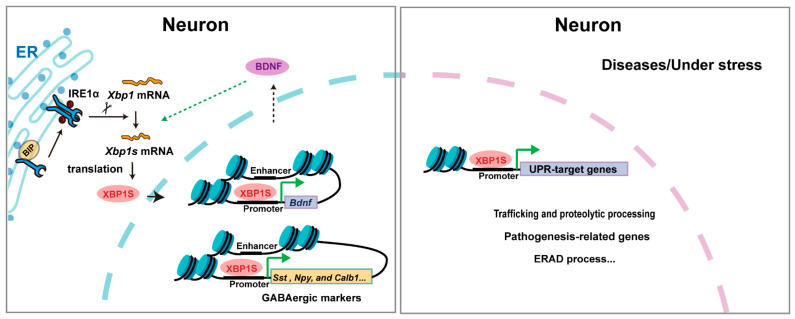
The mechanism of XBP1 in neurons. Triangular arrows: positive regulation or promoting effects.

XBP1 plays multifaceted roles in the nervous system, regulating neuronal differentiation, neurite outgrowth, and synapse formation during development (Figure 3). During this period, *Xbp1s* mRNA is highly expressed, and XBP1S facilitates neurotrophic signaling—particularly of BDNF—between neurites and the nucleus to promote neurite extension [37]. In *Xbp1*-knockout neurons, the upregulation of GABAergic markers such as somatostatin (*Sst*), neuropeptide Y (*Npy*), and calbindin (*Calb1*) is suppressed, impairing BDNF-induced neurite outgrowth [13]. Consistent with a developmental role, nervous system-specific *Xbp1* knockout in mice leads to deficits in hippocampal contextual memory and long-term potentiation, with accompanying cognitive decline [14]. Conversely, constitutive neuronal expression of XBP1S induces recurrent spontaneous seizures and premature lethality [38], indicating that precise regulation of XBP1 activity is critical for normal neural circuit maturation. These findings collectively demonstrate that XBP1’s functions in neural development are largely independent of endoplasmic reticulum (ER) stress. Despite the absence of documented human neurodevelopmental disorders linked to XBP1 mutations, evidence from expression analyses and conditional knockout models underscores its importance in neural development.

Transitioning from development to adult homeostasis, XBP1 continues to exert important functions and is implicated in the pathogenesis of diverse psychiatric and neurodegenerative disorders [39]. Recent research indicates that early senescent neurons confer neuroprotection against age-related decline by transferring heat shock proteins via extracellular vesicles to activate the IRE1α-XBP1 pathway and upregulate chondroitin synthase in glial cells [40]. In neurodegenerative diseases—such as Alzheimer’s (AD), Parkinson’s (PD), Huntington’s (HD), and amyotrophic lateral sclerosis (ALS)—synaptic dysfunction and neuronal death are linked to proteostasis disruption. Here, XBP1 exhibits context-dependent roles: in a C. elegans AD model, UPR activation aggravates Aβ toxicity, whereas XBP1 overexpression in murine AD models reduces aberrant protein aggregation and attenuates disease progression [25,41]. In PD, XBP1 supports dopaminergic neuron survival under both physiological and pathological conditions [26]. Paradoxically, *Xbp1*-deficient mice show slowed disease progression in models of ALS (*Sod1* mutation) and HD [12,27], suggesting a complex, disease-specific involvement. Beyond neurodegeneration, XBP1 influences central metabolic regulation; constitutive expression of XBP1S in POMC neurons—key regulators of energy balance—restores body weight homeostasis in obese mice [29]. While direct evidence from human mutations linking XBP1 to common neurodegenerative diseases is still emerging, its central role in proteostatic and metabolic maintenance highlights its potential therapeutic relevance.

### 2.2. Cardiovascular System

**Figure 4:**
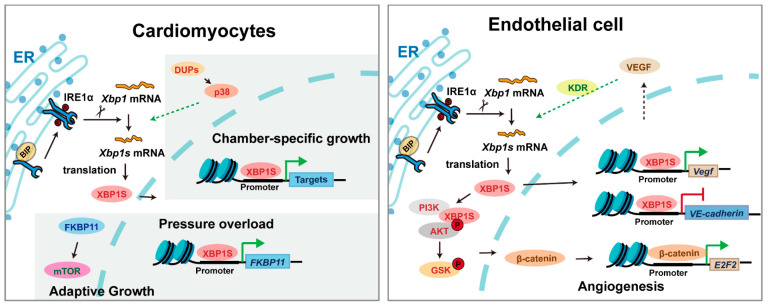
The mechanism of XBP1 in cardiomyocytes and vascular endothelial cells. Triangular arrows: positive regulation or promoting effects; flat-headed arrows: negative regulation or inhibitory effects.

XBP1 serves as a master regulator orchestrating cardiac development and maturation (Figure 4). During cardiac morphogenesis, XBP1 governs critical developmental events—its ablation in mice leads to profound structural defects including ventricular wall hypoplasia, impaired trabeculation, and disrupted septal formation, ultimately resulting in embryonic lethality [15]. Intriguingly, the postnatal heart exhibits chamber-specific XBP1 activation patterns, with predominant left ventricular expression driving its functional maturation and hemodynamic dominance [42]. While cardiomyocyte-specific *Xbp1* knockout mice are viable at birth, they develop progressive contractile dysfunction and premature mortality, revealing XBP1’s essential role in maintaining cardiac homeostasis beyond development [30]. Although direct evidence of *XBP1* mutations causing human congenital heart defects remains limited, its conserved expression and functional necessity across species highlight its potential contribution to cardiovascular developmental pathways.

Beyond development, XBP1 is a critical mediator of cardiovascular adaptation under pathological stress. In conditions such as ischemia, pressure overload, or heart failure, XBP1 is robustly activated and mediates adaptive remodeling through coordinated regulation of hypertrophy, metabolism, and survival pathways. Genetic studies demonstrate its protective role, as cardiac-specific *Xbp1* deficiency exacerbates dysfunction while its activation preserves contractility [30,43]. In the vasculature, XBP1 exhibits context-dependent regulation of angiogenesis—it is indispensable for VEGF-mediated neovascularization during development and ischemic repair [16,44], yet chronic activation triggers excessive autophagy and endothelial apoptosis, paradoxically promoting atherosclerotic progression [31]. These dual roles underscore XBP1’s complex integration of developmental programs and stress responses in cardiovascular biology. Emerging clinical associations suggest that specific *XBP1* polymorphisms may influence susceptibility to hypertension and coronary artery disease in humans, further supporting its translational relevance in cardiovascular homeostasis and pathology.

### 2.3. Immune System

**Figure 5:**
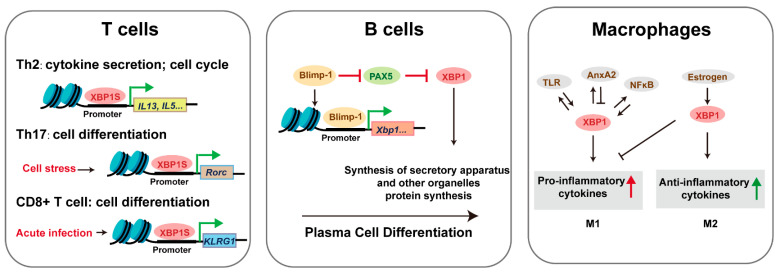
The mechanism of XBP1 in immune cells. Triangular arrows: positive regulation or promoting effects; flat-headed arrows: negative regulation or inhibitory effects.

XBP1 plays a stage-specific role in adaptive immunity, with its functions becoming particularly critical under conditions of cellular stress (Figure 5). While T cell development proceeds normally in *Xbp1* knockout mice, thymic defects are markedly exacerbated when combined with SEL1L deficiency, revealing XBP1’s compensatory role in ERAD-impaired conditions [32,45]. In mature T cells, XBP1 regulates subset differentiation through distinct mechanisms: (1) XBP1S drives Th2 responses via enhanced proliferation and cytokine production [33,46], (2) stress-induced XBP1 promotes Th17 differentiation in autoimmunity [47], and (3) XBP1S enhances CD8+ effector function through upregulation of killer cell lectin-like receptor G1 [48]. Although direct evidence of XBP1 mutations causing human T-cell immunodeficiencies is currently lacking, its conserved role in T-cell stress adaptation suggests potential relevance to immune dysregulation syndromes.

In the humoral arm of immunity, XBP1 is indispensable for terminal B-cell differentiation. It mediates organelle biogenesis required for plasma cell function and antibody secretion [49]. While Xbp1 deficiency permits initial plasmablast formation, it severely impairs bone marrow homing and sustained antibody production [17], whereas forced XBP1 expression significantly boosts immunoglobulin output [50]. NK cells similarly require XBP1 for effective anti-tumor and anti-infection responses. Among innate immune cells, TLR-activated XBP1 splicing serves as a key modulator of inflammatory responses [51]. Macrophages exhibit context-dependent responses to XBP1 activation: transient XBP1S induces autophagy and proliferation, while chronic expression triggers apoptosis [52]. The P300/XBP1S/Herpud1 axis has been identified as a driver of M2 polarization in pathological contexts such as macular degeneration [53]. Additionally, XBP1 deficiency reduces dendritic cell survival—a defect reversible by progenitor cell overexpression [18]—and selectively impairs eosinophil differentiation through disruption of secretory granule formation, without affecting neutrophil or basophil development [19]. These findings across lymphoid and myeloid lineages highlight XBP1 as a central regulator of immune cell development, function, and stress adaptation, with emerging implications for understanding human immune pathologies.

### 2.4. Skeletal System

**Figure 6:**
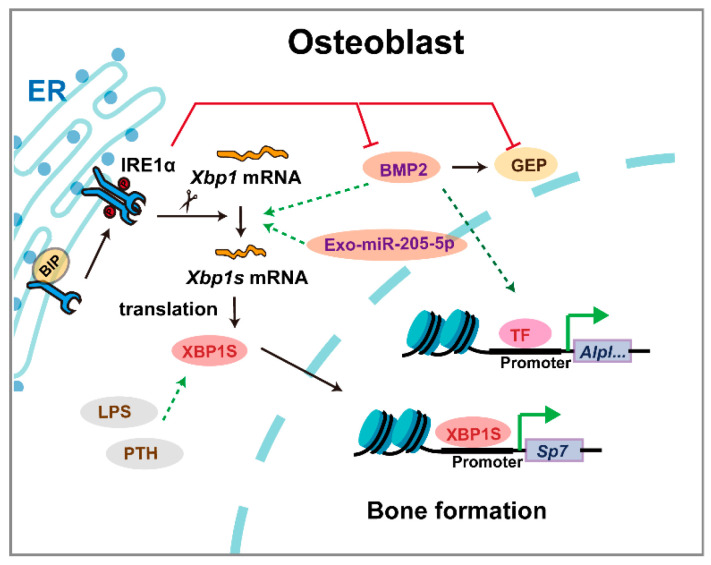
The mechanism of XBP1 in osteoblasts. Triangular arrows: positive regulation or promoting effects; flat-headed arrows: negative regulation or inhibitory effects.

XBP1 plays context-dependent roles in skeletal development, with distinct mechanisms governing chondrogenesis and osteogenesis (Figure 6). During BMP2-induced chondrocyte differentiation, IRE1α activation promotes *Xbp1* mRNA splicing, upregulating granulin precursor protein to facilitate endochondral ossification [54,55]. Chondrocyte-specific *Xbp1* knockout mice exhibit transient dwarfism and chondrodysplasia during development, which normalizes in adulthood despite IRE1α overactivation without triggering decay pathways [21]. Paradoxically, excessive *Xbp1* expression also impairs cartilage development by inducing ER stress-mediated suppression of growth plate chondrocyte proliferation [56]. A regulatory network involving LncRNA/circRNA-miRNA interactions in Xbp1-deficient chondrocytes offers potential therapeutic targets for cartilage disorders [57]. While direct evidence of *XBP1* mutations causing human skeletal dysplasia remains limited, its conserved role in cartilage development suggests potential relevance to chondrodysplasia phenotypes.

Beyond developmental patterning, XBP1 continues to regulate skeletal homeostasis through integrated stress response pathways. In osteoblast differentiation, *Xbp1* deficiency specifically impairs late-stage maturation by suppressing Osx transcription, without affecting early differentiation [20]. IRE1α, XBP1, and BMP2 form a feedback loop fine-tuning osteogenesis [58]. Notably, chaperone protein BIP upregulation ameliorates bone loss in osteoporosis models, underscoring XBP1’s role in maintaining osteoblast viability under stress [59]. Although *Xbp1*^−/−^; *Liv*XBP1 mice complete ossification, mineralization remains deficient [8]. XBP1 also regulates craniofacial homeostasis, with genome-wide analyses identifying it as critical for temporomandibular joint (TMJ) function and regeneration [60]. Furthermore, XBP1 emerges as a shared regulator in fibrocartilage formation and heterotopic ossification, suggesting therapeutic potential for tendon-bone interface healing [61]. These findings establish XBP1 as a key modulator of both skeletal development and maintenance, with implications for understanding and treating various bone and cartilage disorders.

### 2.5. Exocrine Glands

XBP1 is a master regulator of ER biogenesis and chaperone expression, essential for meeting the high protein-folding demands of exocrine glands [62,63]. *Xbp1*^−/−^; *Liv*XBP1 mice exhibit severe postnatal growth retardation and early lethality due to pancreatic hypoplasia and deficient digestive enzyme production [8]. This phenotype is linked to acinar cell apoptosis at E18.5, though XBP1 is dispensable for endocrine pancreas development and function [8]. In salivary glands, *Xbp1* expression increases during acinar cell maturation, and its loss reduces ER volume and disrupts lobular architecture [8,55]. Single-cell RNA-seq confirms XBP1 as a top transcriptional correlate of acinar development [55].

Similarly, the mammary gland—a specialized exocrine organ—relies on XBP1 for structural and functional adaptation during lactation. Epithelial-specific knockout models (*hGFAP-Cre* or *BLG-Cre*; *Xbp1^fl/fl^*) reveal that *Xbp1* loss impairs ductal branching and bud formation in virgin mice, induces stromal fibrosis, and causes chronic ER stress during lactation, suppressing proliferation and differentiation without apoptosis [9,24]. Consequently, *Xbp1*-deficient mice show reduced milk synthesis, impaired pup growth, and increased mortality [9]. Notably, *Xbp1* deletion reduces ER content by pregnancy day 18 and blocks alveolar expansion, leaving persistent adipocytes [24]. These results affirm XBP1 as a central coordinator of mammary epithelial integrity, ER homeostasis, and secretory capacity throughout reproductive stages.

## 3. The Promises and Challenges of XBP1 in Regeneration

XBP1 serves as a master coordinator of regenerative processes across multiple organ systems through distinct yet interconnected mechanisms [7,64]. In skeletal muscle, the IRE1α-XBP1 axis promotes myoblast fusion *via Mymk* activation and satellite cell proliferation, with both genetic and therapeutic studies demonstrating its essential role in muscle repair [7,64]. These findings position XBP1 modulation as a promising therapeutic strategy for treating muscle degenerative diseases such as Duchenne muscular dystrophy and age-related sarcopenia. For cutaneous wound healing, XBP1S enhances tissue regeneration by upregulating critical growth factors (PDGF-BB, TGF-β3) [65], stimulating collagen synthesis through β-catenin signaling [66], and promoting angiogenesis [16,44,67,68]. The demonstrated efficacy of topical XBP1 activators in preclinical models of diabetic ulcers suggests significant translational potential for chronic wound management. The protein similarly drives bone regeneration by enhancing the osteogenic potential of periodontal ligament cells [69] and facilitating chondrogenic differentiation of mesenchymal stem cells [70,71]. This mechanistic understanding provides a foundation for developing XBP1-targeted therapies to accelerate fracture healing and treat osteoporosis. Beyond these established roles, emerging evidence reveals XBP1’s involvement in liver regeneration post-hepatectomy [72], neuroprotection through serotonin pathway modulation [73], and metabolic regulation via obesity reversal and insulin sensitivity restoration [74]. The breadth of these regenerative functions highlights XBP1’s potential as a multi-organ therapeutic target, though tissue-specific delivery systems will be crucial for clinical application.

**Figure 7:**
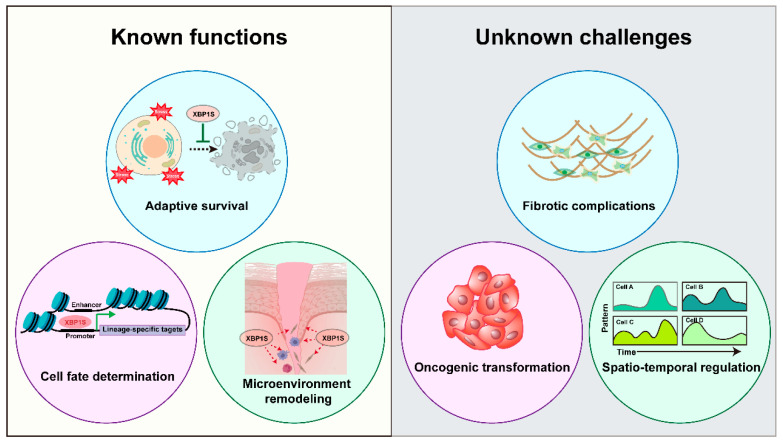
XBP1 in Regeneration: Mechanisms and Challenges. Schematic representation of XBP1’s dual roles in tissue regeneration, illustrating its established functions in adaptive survival, cell fate determination, and microenvironment remodeling, alongside key challenges including fibrotic complications, oncogenic risks, and spatiotemporal regulation requirements.

As illustrated in Figure 7, XBP1 orchestrates multiple regenerative processes through three key mechanisms: enhancing cellular adaptive survival pathways, directing progenitor cell fate decisions, and remodeling the tissue microenvironment to facilitate repair. From a therapeutic perspective, these mechanisms offer multiple intervention points—from small molecule IRE1α/XBP1 pathway modulators to gene therapy approaches—each with distinct clinical implications. While preliminary findings highlight XBP1’s regenerative potential, critical knowledge gaps remain that require systematic investigation. First, tissue-specific mechanistic studies employing inducible genetic ablation and pharmacological modulation are needed to delineate the precise role of the IRE1α/XBP1 pathway across different regenerative contexts. These studies should specifically address optimal therapeutic windows and dosage parameters for potential clinical translation. Second, comprehensive safety assessments must address potential risks associated with sustained XBP1 activation, particularly concerning fibrotic complications and oncogenic transformation [75]. The oncogenic potential of sustained XBP1 activation is a significant concern. For instance, XBP1 has been demonstrated to drive tumorigenesis and progression in multiple myeloma by promoting the survival of malignant plasma cells within the hypoxic bone marrow niche [76]. Similarly, its overexpression is linked to poor prognosis in breast cancer, where it facilitates tumor cell proliferation and chemoresistance [77]. Prolonged XBP1 signaling may also contribute to fibrotic pathologies. Evidence from models of liver fibrosis indicates that the IRE1α-XBP1 pathway is persistently activated in fibroblasts, promoting their activation and excessive extracellular matrix deposition [78,79]. This suggests that uncontrolled XBP1 activation could similarly impede regenerative processes by favoring scar tissue formation over functional tissue restoration. Overcoming these challenges through targeted delivery systems and temporal control of XBP1 activity will be essential for realizing its full therapeutic potential.

Notwithstanding these challenges, XBP1 emerges as a highly promising therapeutic target for regenerative medicine. Future research directions should focus on elucidating spatiotemporal regulation of XBP1 activity during regeneration, developing tissue-selective delivery systems and optimizing activation protocols to maximize regenerative outcomes while minimizing off-target effects. These advances will facilitate translation of XBP1-based therapies into clinical applications for diverse regenerative indications.

## 4. Conclusions

XBP1 has emerged as a master transcriptional regulator with profound implications for both developmental biology and regenerative medicine. This review systematically synthesizes current understanding of XBP1’s multifaceted roles, elucidating its molecular mechanisms across diverse physiological contexts. Our analysis reveals XBP1’s dual functionality as a crucial developmental modulator governing tissue morphogenesis, and a potent mediator of regenerative processes through stress adaptation and cellular reprogramming. The translational potential of XBP1 is particularly noteworthy, with emerging evidence supporting its therapeutic targeting for enhanced tissue repair. Future investigations should focus on three key areas: first, delineating tissue-specific regulatory networks controlled by XBP1; second, developing precision modulation strategies to harness its regenerative capacity; and third, addressing safety considerations for clinical translation. These efforts promise to advance both fundamental understanding of developmental processes and innovative approaches for treating degenerative diseases and traumatic injuries. Synergistic application of single-cell multi-omics platforms and next-generation organoids promises to unveil the precise spatiotemporal control mechanisms of XBP1 in human tissue regeneration.

## Figures and Tables

**Table 1:** The role of Xbp1 in the development of different systems.

System	Tissue/Cell Type	Developmental Model	Phenotype	ER Stress Dependency	Targets	References
Nervous System	Neuronal Cells	Neurons from *Xbp1^−/−^* mice	Impaired neurite outgrowth	No	*Sst, Calb1, Npy*	[13]
	Hippocampus	*Nestin-cre*; *Xbp1^fl/fl^* mice	Cognitive dysfunction	No	*Bdnf*	[14]
Circulatory System	Heart	*Xbp1^−/−^* mice	Severe defects in heart development	No	Not mentioned	[15]
	Endothelial Cells	*CAG-cre*; *Xbp1^fl/fl^* mice	Reduced embryonic vasculature, growth retardation	No	Not mentioned	[16]
	Endothelial Cells	*Tie2-cre*; *Xbp1^fl/fl^* mice	Delayed early retinal angiogenesis	No	Not mentioned	[16]
Immune System	B Cells	*MD4-cre*; *Xbp1^fl/fl^* mice	Form plasmablasts but fail to colonize in the bone marrow and maintain antibody production	No	Not mentioned	[17]
	Dendritic Cells	*Xbp1/Rag2^−^*^/*−*^ chimera mice	Reduced numbers and viability of conventional and plasmacytoid dendritic cells	No	Not mentioned	[18]
	Hematopoietic Cells	*Vav1-cre*; *Xbp1^fl/fl^* mice	Complete loss of eosinophils, no effect on peripheral basophils or neutrophils	No	*Gata1, Prg2, Epx*	[19]
	Eosinophils	*Epx-cre*; *Xbp1^fl/fl^* mice	Reduced eosinophil numbers	No	Not mentioned	[19]
Skeletal System	Osteoblasts	In vitro osteoblast differentiation model using *Ire1α^−/−^* cells	Inhibition of osteoblast maturation	No	*Osx*	[20]
	Chondrocytes	*Col2a1-cre*; *Xbp1^fl/fl^* mice	Chondrodysplasia	No	Not mentioned	[21]
Digestive System	Intestinal Epithelial Cells	*Villin-cre*; *Xbp1^fl/fl^* mice	ER stress and spontaneous enteriti	No	Not mentioned	[22]
	Liver	*Xbp1^−/−^* mice	Severe liver hypoplasia	No	Not mentioned	[23]
	Salivary Glands	*Xbp1^−/−^*; *Liv*XBP1 mice	Poor ER development in acinar cells, increased intercellular spaces	Yes	Not mentioned	[8]
	Pancreas	*Xbp1^−/−^*; *Liv*XBP1 mice	Pancreatic hypoplasia and impaired production of pancreatic digestive enzymes	Yes	Not mentioned	[8]
Others	Mammary Epithelial Cells	*hGFAP-cre*; *Xbp1^fl/fl^* mice	Poor branching morphogenesis, impaired terminal bud formation, spontaneous stromal fibrosis	Yes	Not mentioned	[9]
	Mammary Epithelial Cells	*BLG-cre*; *Xbp1^fl/fl^* mice	Low ER abundance, insufficient alveolar expansion	Yes	Not mentioned	[24]

**Table 2:** The role of Xbp1 in tissue homeostasis.

System	Tissue/Cell Type	Disease Model	Phenotype	ER Stress Dependency	Targets	References
Nervous System	Brain	Alzheimer’s disease transgenic mice + AAV2 *Xbp1s*	Improved synaptic function and protein homeostasis	No	*Cofilin-1*	[25]
	Substantia Nigra	*Nestin-cre*; *Xbp1^fl/fl^* mice	Spontaneous neurodegenerative signs	Yes	*Calreticulin, ERp72*	[26]
	Substantia Nigra	Parkinson’s disease mice + AAV *Xbp1s*	Increased survival of dopaminergic neurons	Yes	Not mentioned	[26]
	Nervous System	*Nestin-cre*; *Xbp1^fl/fl^* mice + experimental amyotrophic lateral sclerosis model	Enhanced clearance of misfolded protein aggregates, resistance to disease progression	Yes	*Edem1*	[12]
	Brain	*Nestin-cre*; *Xbp1^fl/fl^* mice + Huntington’s disease model	Increased secretion of soluble mutant huntingtin, resistance to disease progression	Yes	*Igf2*	[27]
	Hypothalamus	*Nestin-cre*; *Xbp1^fl/fl^* mice + high-fat diet	Obesity, hypothalamic leptin resistance	Yes	Not mentioned	[28]
	Pomc Neurons	Pomc neuron-specific induced expression of *Xbp1s* mice + high-fat diet	Improved insulin sensitivity and glucose levels, prevention of diet-induced obesity	Yes	Not mentioned	[29]
Circulatory System	Cardiomyocytes	*αMHC-cre*; *Xbp1^fl/fl^* mice	Cardiac contractile dysfunction in adulthood, shortened lifespan	Yes	*Fkbp11*	[30]
	Cardiomyocytes	*αMHC-cre; Xbp1^fl/fl^* mice + hypertension condition	Cardiac dysfunction, heart failure	Yes	*Fkbp11*	[30]
	Cardiomyocytes	*αMHC-cre*; *Xbp1^fl/fl^*; *αMHC-tTA*; *TRE-Tg Xbp1s* mice + hypertension condition	Restored cardiac adaptive growth, prevented cardiac dilation and heart failure under hypertension	Yes	*Fkbp11*	[30]
	Endothelial Cells	*Tie2-cre*; *Xbp1^fl/fl^* mice + ischemia model	Impaired angiogenesis in ischemic muscle tissue	Not mentioned	Not mentioned	[16]
	Endothelial Cells	Endothelial cells + Ad *Xbp1s*	Increased autophagy	No	*Beclin-1*	[31]
Immune System	Thymocytes	*CD2-icre*; *Sel1 ^fl/fl^; Xbp1^fl/fl^* mice	More severe thymocyte developmental defects	Yes	Not mentioned	[32]
	CD4^+^ T Cells	*CD4-cre*; *Xbp1^fl/fl^* mice + airway allergy model	Prevented induction of Th2 cell polarization in the airway	No	*Il4*	[33]
Digestive System	Intestinal Epithelial Cells	*Villin-cre*; *Xbp1^fl/fl^* mice + experimental colitis model	Exacerbated inflammation and bleeding	Yes	Not mentioned	[22]
	Hepatocytes	Adult AAV8-*Transthyretin-cre*; *Xbp1^fl/fl^* mice + high-fructose diet	Acute liver injury	Yes	Not mentioned	[34]
	Hepatocytes	*Alb-cre*; *Xbp1^fl/fl^* mice + high-fat diet	Inhibited development of steatohepatitis	Yes	Not mentioned	[35]

## Data Availability

No new data were created or analyzed in this study. Data sharing is not applicable to this article as it is a comprehensive review of previously published literature.

## Abbreviations

The following abbreviations are used in this manuscript:

XBP1	X-box binding protein 1
IRE1α	Inositol requiring enzyme 1
ER	endoplasmic reticulum
bZIP	basic leucine zipper
UPR	unfolded protein response
NES	nuclear export signal
ERAD	ER-associated degradation
AD	Alzheimer’s disease
PD	Parkinson’s disease
HD	Huntington’s disease
ALS	amyotrophic lateral sclerosis
TMJ	temporomandibular joint
